# Meta-analysis of soil mercury accumulation by vegetables

**DOI:** 10.1038/s41598-018-19519-3

**Published:** 2018-01-19

**Authors:** Haixin Yu, Jing Li, Yaning Luan

**Affiliations:** 1 College of Forestry, Beijing Forestry University, No. 35, Qinghua East Road, Haidian District, Beijing, 100083 PR China; 2 Zhengzhou Hualiang Technology Co., Ltd, No.28 Shangwu Inner Ring Road, Zhengdong New Area CBD, Zhengzhou, 450046 PR China; 3 School of Life and Environmental Science, University of Sydney, Biomedical Building, Locomotive St, Eveleigh, Sydney, NSW 2015 Australia

## Abstract

Mercury pollution in soil poses serious risks to human health through consumption of contaminated vegetables. We used a meta-analysis to examine the mercury enrichment ability of different vegetables and the main factors affecting mercury uptake. We drew the following conclusions. (1) Plants with a lower bioconcentration factor (BCF) include cowpea, long bean, and radish, whereas plants with a higher BCF include green pepper, spinach, cabbage, and Chinese cabbage. (2) Leaf and cucurbit have the highest and lowest capacity, respectively, for mercury enrichment. (3) When soil pH is <6.5, mercury level uptake by the plant increases, whereas it decreases when the pH is >7.5, meaning that increased soil pH reduces mercury uptake in soil. (4) When soil organic matter (SOM) is lower than 20 g/kg, tuber plants have the highest and eggplant has the lowest mercury adsorption capacity, respectively. When SOM is 20–30 g/kg, cucurbit has the lowest and leaf the highest adsorption capacity, respectively. When SOM is higher than 30 g/kg, however, eggplant has the highest mercury adsorption capacity, but there were no significant differences among the five types of vegetables. We argue that this meta-analysis aids in selecting vegetables suitable for absorption of heavy metals from polluted soil.

## Introduction

Mercury (Hg) is liquid in standard ambient temperature conditions (25 °C), existing as elemental mercury in the atmosphere, soil, and water in a zero oxidation state. At this temperature, mercury easily evaporates from contaminated floors, walls, or clothing, thus becoming a source of secondary air pollution. The Hg in soil has three valence states: 0, +1, and +2. The Hg in soil is classified as soluble in the presence of free ions or soluble compounds. In the soil environment, Eh and pH determine the Hg valence. In soil Hg^2+^ in reductive conditions contain HS, generate insoluble HgS, when soil oxygen is sufficient, HgS can be slowly oxidized to Hg_2_SO_4_ and HgSO_4_^1^. The unique physical and chemical properties of Hg mean that it is widely used in industrial chemical applications, paper manufacturing, mining, and defense industries. For example, chemical wastewater containing mercury is discharged into the surrounding soil; some of the mercury ions are then adsorbed onto the soil, and industrial waste gas is discharged through dry and wet deposition, causing serious soil pollution.

Five heavy metals (HMs) (Cd, Pb, Ni, Hg, and As) promote vegetable growth at low concentrations, but inhibit growth at higher concentrations^2^. A previous study reported that the consumption of plants found in areas where HMs are present in the soil causes serious damage to metabolic functions^3^. HMs also damage the digestive tract and kidneys, with both inorganic and organic Hg playing leading roles in causing these harmful effects. Common forms of inorganic Hg, such as HgS and HgCl_2_, can enter the body through food or simply by inhalation^4^. The presence of small amounts of methyl Hg in pregnant women may cause miscarriage or stillbirth. When pregnant women have increased mercury intake, the fetus may show symptoms of mental bradypsychia, or may even be at risk of congenital Minamata disease^5^. Stigliani has described the delayed effects of Hg and its serious role in contributing to environmental pollution as a “chemical time bomb”^6^.

Searching for evidence of HM adsorption in soil and undertaking risk assessments of the effects of HM on human health have both recently become important issues. The widespread nature of pollution and wide range of species affected have led to studies on adsorption capacity for a large number of vegetables intended for human consumption^7,8^. Crops show considerable differences in the way they adsorb HMs because of the variation of plant growth traits, genetic characteristics, physiological properties, morphological and anatomical features, and ion transport mechanisms^9–12^. Research into the effects of different vegetable varieties on soil Hg adsorption capacity has an important role in making it feasible to control the human intake of HMs.

Currently, most suburban soils of cities of our country demonstrate varying degrees of Hg pollution, and in many local vegetables, fruits, and other foods, the heavy metal content exceeds the standard or is close to the critical value. In China, large cities such as Beijing, Shanghai, Tianjin, Guiyang, Datong, Bengbu, Chengdu, Harbin, Fuzhou, and Shouguang, and medium-sized cities such as Changsha systematically showed heavy metal pollution in suburban vegetable garden soils and vegetables in a survey. The qualified soil quality standards stipulated by the state laws of China are as follows. When pH < 6.5, Hg ≤ 0.25 mg/kg; when pH = 6.5–7.5, Hg ≤ 0.30 mg/kg; and when pH > 7.5, Hg ≤ 0.35 mg/kg^13^.

In addition to the species-specific differences in the manner in which soil Hg is taken up by different plants, precipitation can, for example, also alter this process by diluting the mercury content in soil, thus affecting the adsorption of mercury by the plant. Therefore, there is benefit in employing statistical methods to summarize and analyze data in existing literature about the accumulation of HM pollutants in vegetables. As most studies summarize existing data, to the best of our knowledge, no study has used statistical methods to integrate and evaluate research on this topic to date. Meta-analysis consists of multiple independent experiments towards a common purpose for quantitative research, combined analysis, statistical methods, and a summarized evaluation. In the present study, we conducted a meta-analysis to summarize the Hg adsorption capacity of vegetables, including those grown in China and other countries, to: (1) analyze and compare Hg adsorption capacities of different vegetables to provide a reference for future research, and (2) provide reasonable suggestions for vegetable farming by taking advantage of the varying Hg adsorption trends of different types of vegetables.

## Methods

### Documentation indexing

We collected data from the China National Knowledge Infrastructure (CNKI), Springer, Elsevier, and PubMed. First, we identified a number of key words, including “vegetables,” “plants,” “mercury/Hg,” “absorption of mercury/Hg,” “uptake of mercury/Hg,” “heavy metal,” and “meta-analysis.” In addition, we only consulted complete articles from which all data were made available for analysis. We did not include unpublished data or articles that were only summaries of previous literature. Our time frame for studies about the Hg content of vegetables in soil covered the last 10 years (December 2005–December 2015).

### Research and data selection

#### Inclusion criteria

The 27 studies each: (1) included at least one of 24 types of selected plants that are able to absorb concentrations of Hg or bioconcentration factor (BCF); (2) had similar literature research methods in that they exhibited data integrity and included specific information on soil physical and chemical properties; and (3) can be aggregated with the results for statistical indicators of the corresponding expression. For duplicate data, we selected only the most recent and largest set of data.

#### Data filtering and elimination

The following information was selected from each article: (1) first author name, year in which the experiment took place, experimental site; (2) measure of vegetable concentration in soil; (3) the physical and chemical properties of the soil (soil pH, soil organic matter (SOM)) for both experimental group and control group (soil and vegetable samples).

In all, 224 references were collected. We reviewed and evaluated each study, eliminating duplicate reports and studies with poor study quality, low information availability, and incomplete data. We finalized a set of 125 data points for 24 different plants, extracted from the 27 studies, which were included in the final determination (Table 1). Data were analyzed using STATA 12.0 software and Review Manager 5.3 software. Only one set of data could not be compared and was therefore excluded from the analysis. Exclusion criteria for literature were: repeated published literature and incomplete data in literature, or data could not be analyzed.

**Table 1:** Statistics from 27 published articles.

No.	Year	Experimental Site	Types of Vegetables	pH	SOM(g/kg)	References
1	2015	Fujian Province, China	celery, lettuce, shepherd’s purse, ginger, broccoli, pakchoi, cabbage	5.68	30.86	^29^
2	2013	Zhuzhou, Hunan Province zinc smelter, China	celery, lettuce, Chinese cabbage, cabbage	5.78	27.3	^30^
3	2015	Guangdong Province, China	spinach, carrot, cabbage, scallion	5.44	28.4	^31^
4	2012	Guangdong Province, China	pakchoi, carrot	5.21	27.4	^32^
5	2014	Zunyi, Guizhou Province, China	Pepper	6.33	21.86	^33^
6	2014	Estarreja Chemical, Portugal	cabbage, tomato, long bean	6.4	23.9	^34^
7	2007	Beijing, China	carrot, turnip	7.6	13	^35^
8	2006	Guiyang, Guizhou, Province, China	celery, lettuce, tomato, cucumber, leek, eggplant	6.1	31.9	^36^
9	2009	Guilin, Guangxi Province, China	pakchoi	6.99	23.1	^37^
10	2015	Antioquia, Columbia, USA	long bean	6.82	28.37	^38^
11	2015	Shanxi Province, China	celery, Chinese cabbage, spinach, tomato, cabbage, cucumber, leek, eggplant	8.6	15.95	^39^
12	2011	Zhejiang Province, China	celery, Chinese cabbage, leek	7.61	26.6	^40^
13	2008	lead–zinc mine, Hungary	carrot, tomato, longbean, peabean, cucurbita pepo, onion	6.47	21.4	^41^
14	2008	Beijing, China	cabbage	6.9	21.8	^42^
15	2014	Xining, Qinghai Province, China	hot pepper, pakchoi, Chinese cabbage, spinach, carrot, tomato, cucumber, turnip, potato	8.16	47.29	^43^
16	2008	Almaden, Spain	eggplant	5.7	30.61	^44^
17	2005	Huludao, Liaoning Province, China	celery, shepherd’s purse, Chinese, cabbage, spinach, carrot, scallion, tomato, leek, turnip, eggplant, romaine lettuce, long bean, pea bean, cucurbita pepo	5.6	28.1	^45^
18	2012	Western Saudi, Arabia	spinach, carrot, tomato, cabbage, cucumber, turnip, long bean, peabean, onion	6.63	31.32	^46^
19	2009	Beijing, China	carrot	7.6	18.2	^47^
20	2014	Chongqing Province, China	Chinese cabbage, romaine lettuce, potato, long bean	4.57	22	^48^
21	2005	Zhongshan, Guangdong Province, China	Chinese cabbage, water spinach, romaine lettuce	5.74	25.3	^49^
22	2014	Nanjing, Jiangsu Province, China	lettuce, Chinese cabbage, water spinach, romaine lettuce	6.79	31.6	^50^
23	2006	Para, Brazil	cabbage	8.3	17.3	^51^
24	2010	Kuala Selangor, Malaysia	spinach, cucumber, eggplant, long bean, pea bean	7.7	23.5	^52^
25	2010	Sindh, Pakistan	spinach, onion, potato, turnip, cucumber, pumpkin, eggplant, cabbage, broccoli, long bean, tomato	6.2	29.7	^53^
26	2013	Kampong cham, Cambodia	cabbage, carrot, Chinese radish, cucumber, eggplant, long bean, mustard green, sponge gourd	5.69	30.1	^54^
27	2015	Bogra, Bangladesh	broccoli, potato	6.82	24.9	^55^

### Statistical analysis

#### Combined analysis of effect size

The Hg content in vegetables is affected by the Hg content in the soil; the selected literature used different metrics for quantifying the Hg content of both vegetables and soil, and therefore, we incorporated BCF as a comparison index. BCF refers to the ratio of the equilibrium concentration of pollutants in the living body and the pollution concentration in the external environment (BCF = pollutant concentration in vegetables\pollutant concentration in soil)^14^.

The reaction ratio of BCF is calculated as the effect size (ES), and ES is the difference between two groups. ES has no units, which facilitates the comparison of data in different independent experiments.

The calculation formula for the reaction ratio is:

$${\rm{ES}}={\rm{in}}\,{\rm{BCF}}=\,\mathrm{ln}({{\rm{x}}}_{{\rm{e}}}/{{\rm{x}}}_{{\rm{c}}})={{\rm{lnx}}}_{{\rm{e}}}-{{\rm{lnx}}}_{{\rm{c}}}$$

where X_e_ is the Hg content of the vegetables being studied in the experimental group, and X_c_ refers to the control group (i.e., Hg content in soil) value corresponding to X_e_ from the same publication. In this paper, Review Manager 5.3 software was used to combine the effect value of the data and to perform heterogeneity testing. The Q test was used to determine the homogeneity effect. To calculate values of I^2^, a quantity that offers a metric of consistency across trials in a meta-analysis, and those of *P*, if *P* < 0.05, we looked for the existence of heterogeneity and used a random effects model. Otherwise, a fixed effects model was used to calculate combined effects over a 95% confidence interval (95% CI), and a forest map was drawn.

#### Subgroup analysis and meta-regression

Subgroup analysis was employed to assess the impact of various factors. Part of the larger heterogeneity analysis was combined with factors that may have led to heterogeneity of data in a meta-regression. STATA 12.0 software was used to build a regression model through analysis of the influence of the *t-*value and *P*-value to determine heterogeneity of variables. Statistical significance was assumed at *P* < 0.05.

#### Publication bias analysis

Meta-analysis was employed to revise and eliminate publication bias using funnel plots and the Begg method. The funnel plot shows the symmetrical distribution of scattered points. A shearing method was used to eliminate outliers, or other abnormal values, and to fix missing parts along the central part of the funnel plot and both sides of the center^15^.

#### Sensitivity analysis

A sensitivity analysis was used to combine the data and to observe the effect size of the combined values of ES; a 95% confidence interval was used to estimate whether statistically significant changes were produced.

In addition, after exclusion of all individual studies from the meta-analysis, the results changed in a statistically significant way. We eliminated studies that produced greater heterogeneity, and results that did not produce a statistically significant change. This allowed us to analyze the combined data from all individual studies. The merged analysis was a meta-analysis.

## Results

### Comparison of BCF of different vegetables

**Figure 1:**
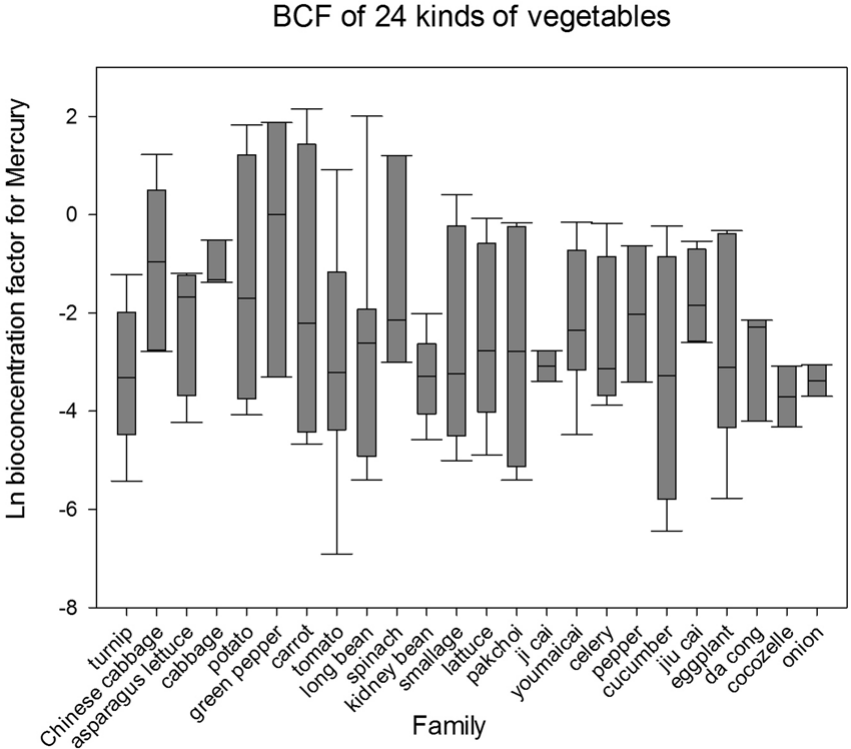
Bioconcentration factor (BCF) of different types of vegetables.

We extracted and calculated 125 data points that satisfied the filtering and elimination criteria for 24 types of vegetables (*n* = 782, mean = −2.3090, max = 2.1518, min = −6.9078). Figure 1 shows that the BCF (a converted value is included following the BCF) for cowpea (*n* = 21, mean = −3.3440, max = −2.0152, min = −4.5854), long bean (*n* = 41, mean = −3.0359, max = 0.9163, min = −6.9078), turnip (n = 47, mean = −3.2529, max = −1.2208, min = −5.4262) was lower than the national BCF standard, i.e., −2.7076. However, green pepper (*n = *17, mean = −0.4777, max = 1.8718, min = −3.3050), spinach (*n* = 9 mean = −0.4784 max = 1.1939 min = −2.1507), cabbage (n = 45 mean = −0.9458 max = −0.5172 min = −1.3744), and Chinese cabbage (*n* = 95 mean = −0.9974 max = 1.2238 min = −2.7855) each had a higher BCF.

According to previous studies^16,17^, in addition to absorption of Hg by plant roots, leaves also absorb Hg from the atmosphere. Therefore, it is inferred that plants can absorb mercury from both the soil and atmosphere. The season can also affect the BCF of plants. Studies have shown that with the change in seasons, the plant root system can change its accumulation ability for heavy metals. However, the effect of the season on vegetables was determined based on the collected statistics. The season can also affect the speciation of heavy metals in soils, such as those in exchangeable or organic-bound states. The effect of the process, which can affect the behavior of soil chemistry, soil properties and surface morphology of Hg, can determine the soil adsorption of Hg. Therefore, the level of plant uptake of Hg in soil, in addition to plant genetic features, is also affected by the physical and chemical properties of soil. The soil chemical behavior of heavy metal ion adsorption is mainly affected by soil pH, organic matter content, and cation exchange capacity, affecting the effective concentration of Hg and thereby indirectly affecting the concentration of Hg uptake from the soil^18^. However, there have not been many descriptions of soil type.

**Figure 2:**
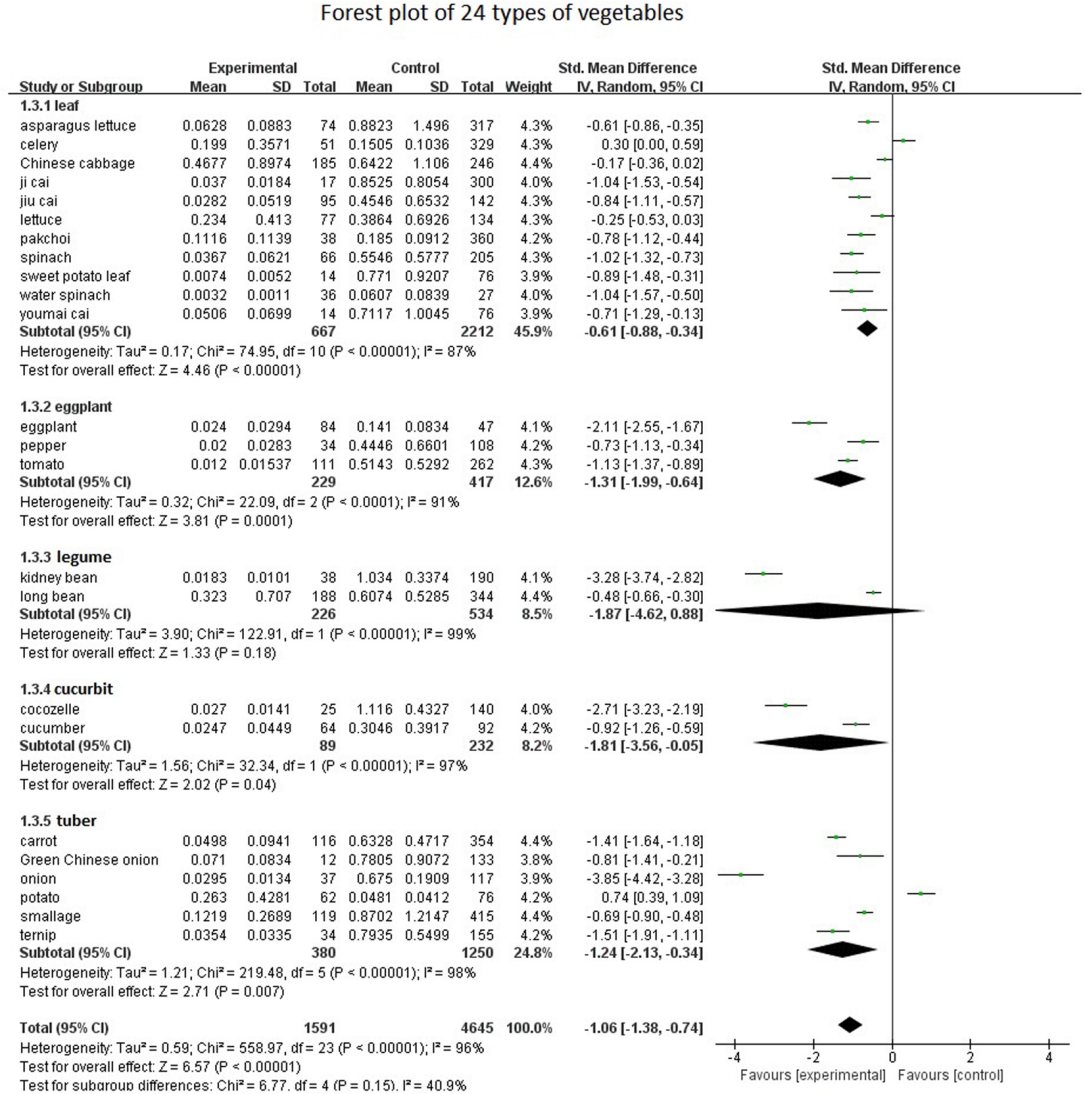
Forest plot of 24 types of vegetables.

We performed a meta-regression of these 24 types of plants (Fig. 2). A meta-analysis of random effects models combined with analysis (SMD = −1.06, 95% CI: −1.38, −0.74, I_2_ = 40.9, *P* = 0.15) was performed. Tau^2^ = 0.59, *P* < 0.05 indicates heterogeneity in statistics. As n > 30, we used *Z-*values. Z = 6.57 and the diamond and vertical lines did not intersect, from which we determined the combined value to have statistical significance. Statistical significance refers to the study of data that can be used in a meta-analysis for statistical analysis and comparison. In a subgroup analysis of 24 vegetables and eggplant, and leaf, the class Tau^2^ decreased, indicating a decrease in heterogeneity. However, the heterogeneity of rhizomes, tuber and cucurbit increased.

### Publication bias

**Figure 3:**
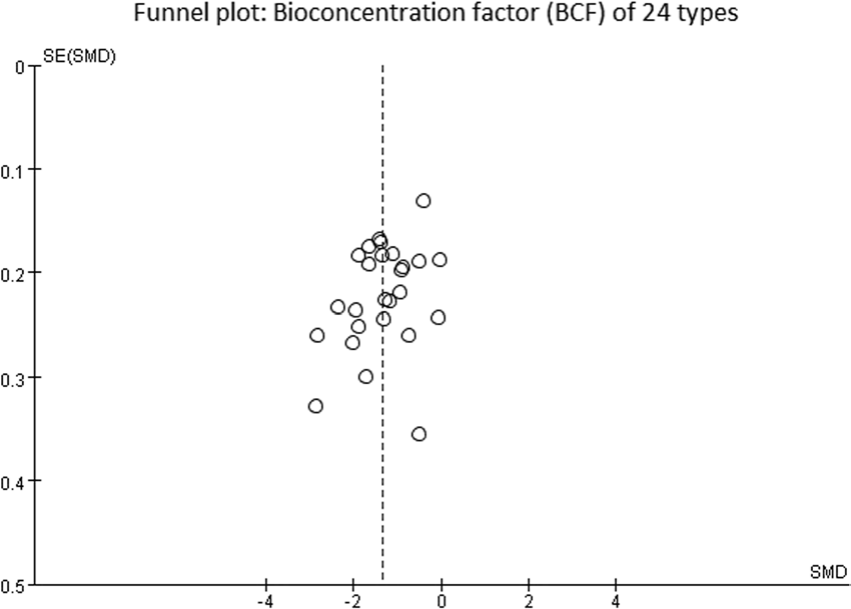
Funnel plot: Bioconcentration factor (BCF) of 24 types of vegetables.

Publication bias occurs when a study with statistically significant findings is more likely to contribute and be published than studies with statistically insignificant findings. Through investigation of the funnel plots, we found a distribution of average symmetry on both sides, indicating that the published data showed bias (Fig. 3).

### Factors that affect the adsorption capacity of vegetables

#### Different vegetable types

**Figure 4:**
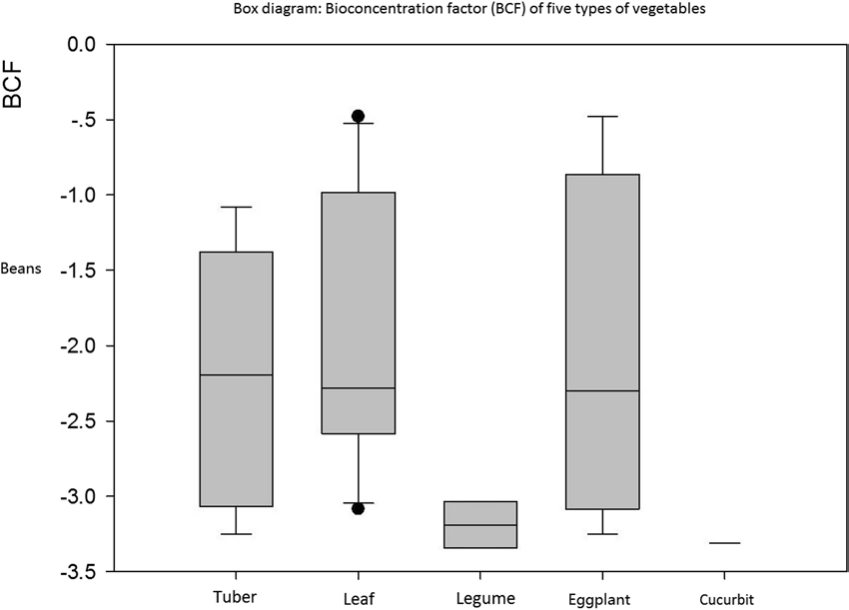
Box diagram: Bioconcentration factor (BCF) of five types of vegetables.

Effect sizes of the same types of vegetable were combined to calculate means and standard deviations. We calculated the BCF of these five different types of vegetables across a confidence interval in a box plot (Fig. 4)^19^.

Average enrichment coefficients for leafy vegetable, bean, eggplant, cucurbit, and root vegetable class were −1.9467, −3.1900, −2.0832, −3.3099, and −2.2170, respectively. This shows that of the five types of vegetables, the ability of leaf and cucurbit to accumulate Hg was the strongest and weakest, respectively. This is consistent with the findings of Chen *et al*.^20^. Overall, absorption levels of HM in leaf were significantly higher than those for eggplant and other vegetables. He^21^ reported similar results. However, other differences exist among the five types of vegetables, similar to the findings of Li^22^. In other studies, mercury enrichment capacity was low in cucurbit, whereas the enrichment coefficient was high in leaf; this is consistent with our results^23^.

#### Soil pH

The adsorption and desorption of HMs in soil is an important process that can affect the chemical behavior and surface properties of soil. Soil surface properties and the morphology of Hg can determine the adsorption status of Hg. After soil adsorption, the chemical behavior of HM ions is mainly affected by soil pH, the influence of SOM effective concentration, cation exchange capacity (CEC), and Hg. The chemical behavior of HMs also indirectly affects plants through soil absorption of Hg. Soil pH is the most important factor influencing the effectiveness of HM absorption; under acidic conditions, the lower pH of soil containing H^2+^ results in greater release of Hg, and Hg activity is enhanced^18^. Therefore, when soil pH is <6.5, adsorption of organic pollutants by soil particles can return pollutants to the soil water. Hg compounds are absorbed by plant roots, resulting in elevated levels of Hg in plants.

**Figure 5:**
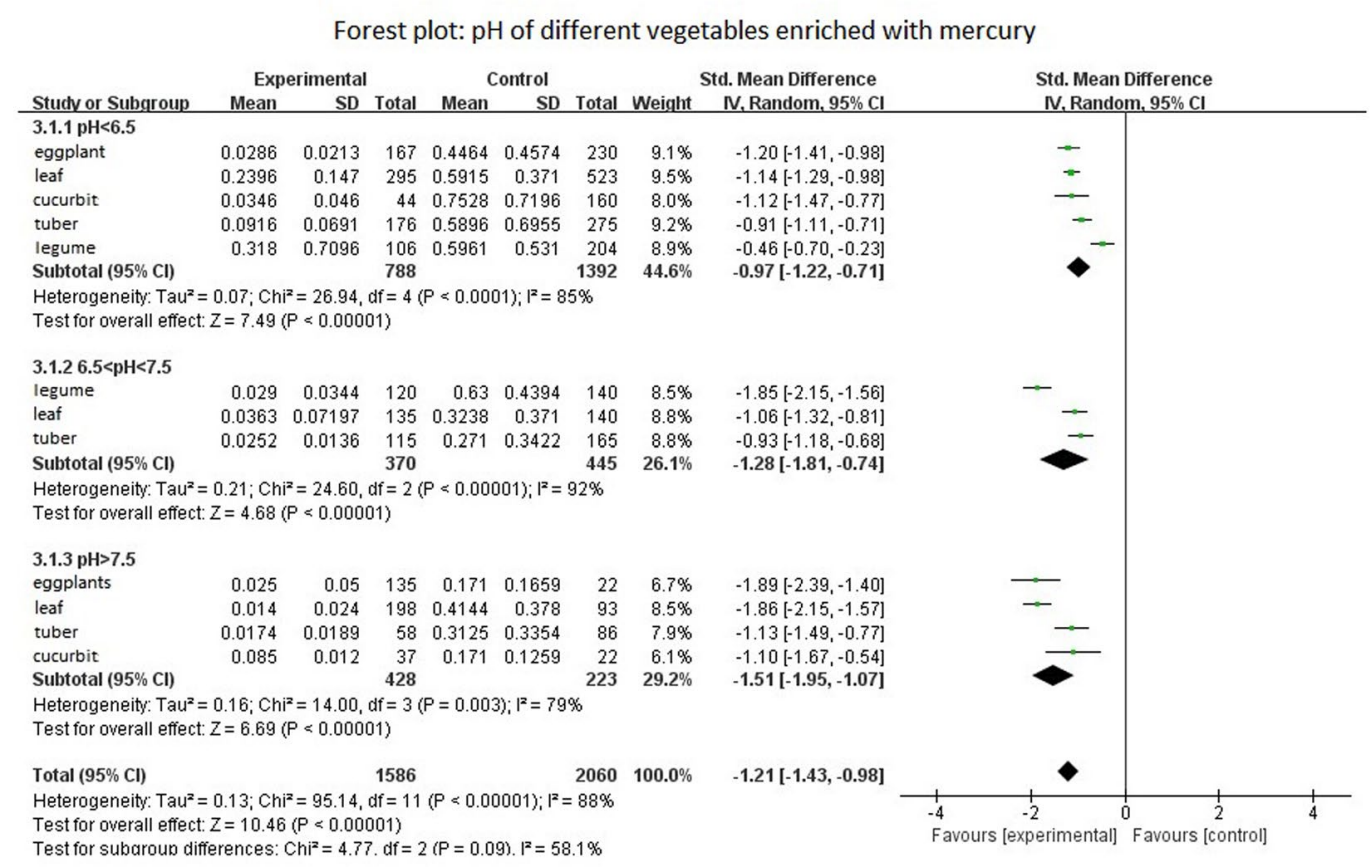
Forest plot: pH of different vegetables enriched with mercury.

In subgroup analyses of different plant pH values, a random effects model combined with analysis; with SMD = −1.21, 95% CI: −1.43 and −0.98, I^2^ = 58.1, *P* = 0.009) revealed that Tau^2^ = 0.13 and *P* < 0.05, indicating data heterogeneity. Z = 7.61 can determine whether the value of the merger has statistical significance. When pH < 6.5, plants absorb somewhat elevated levels of Hg; at pH > 7.5, the level of Hg uptake by plants decreased. A previous study^24^ found similar results, and pH and Hg BCF showed a significant negative correlation. A forest map of different pH values is shown in Fig. 5. Under acidic conditions, the adsorption of Hg^2+^ in soil was higher because the hydrogen and oxygen forms of Hg are more easily adsorbed by SOM than the HgCl_2_ forms. Consequently, soil Hg^2+^ content increases adsorption. When pH levels continue to rise, Hg^2+^ adsorption capacity is gradually reduced, and levels of soil minerals such as kaolinite, spot removal stone, hydrous iron oxide, and silicon dioxide, together with their absorption of Hg^2+^, all begin to decrease. Soil adsorption of Hg^2+^ also decreases^25^.

#### SOM

**Figure 6:**
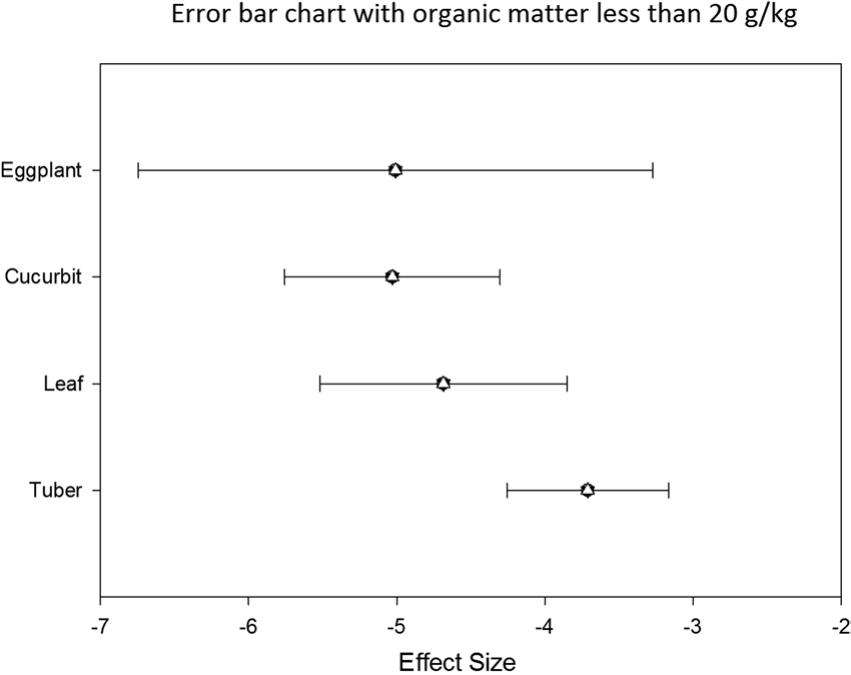
Error bar chart with organic matter less than 20 g/kg.

**Figure 7:**
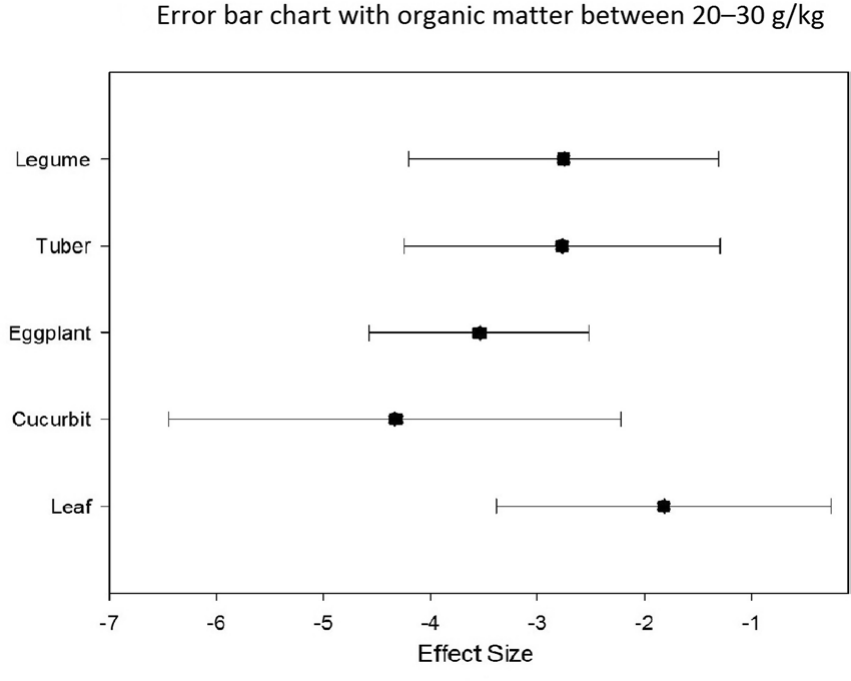
Error bar chart with organic matter between 20–30 g/kg.

**Figure 8:**
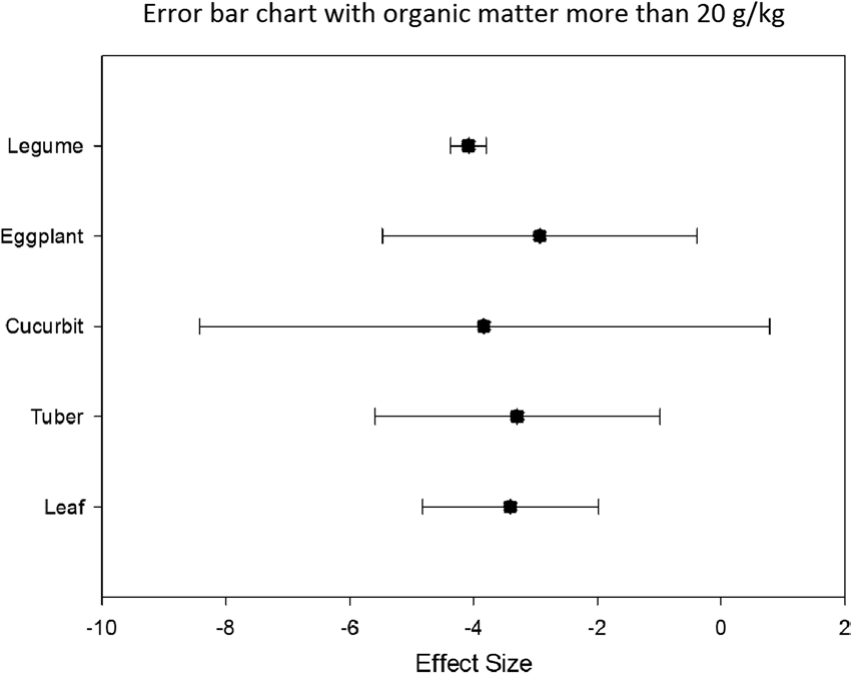
Error bar chart with organic matter more than 20 g/kg.

SOM is one of the main factors affecting the bioavailability of organic pollutants. Analysis of the graph of the five types of vegetables showed that when SOM is <20 g/kg (Fig. 6), the ability of tuber to adsorb Hg is strongest. When SOM is 20–30 g/kg (Fig. 7), the enrichment ability of cucurbit is the lowest, and the enrichment ability of leaf is the highest. When SOM is >30 g/kg, the adsorption capacity of the five vegetables is insignificant (Fig. 8).

Several studies have shown that soil inorganic colloids adsorb organic Hg, and the organic compound is an inorganic Hg adsorber. According to Johanson, there are two points governing the Hg adsorption mechanism of organic matter: first, under normal circumstances, soil organic matter has a stronger affinity than inorganic compounds; second, soil organic matter particles have a greater surface area than the inorganic compound^26^. The adsorption of soil particles on heavy metal organic pollutants reduces the direct flow of pollutants into the soil water. Plants absorb contaminants from the soil water content of Hg, which is higher in plant roots than in other parts. Soil moisture can inhibit the soil particle surface adsorption ability of pollutants and improve its bioavailability; however, when there is too much soil water, plants will undergo oxygen shortage, tuber formation, and a weakening of the absorption of pollutants. Thus, when the soil organic matter content is higher, the absorption of inorganic Hg compounds in soil, and the vegetable soil uptake of mercury, are also higher.

#### Atmospheric factors

In this study, vegetables were discussed with respect to their absorption classification (leaf, eggplant, legyme, cucurbit, and tuber), and the surrounding soil pH and SOM in which these vegetables were planted. The mercury concentration in vegetables was determined to be closely related to that in the atmosphere.

In addition to absorbing Hg from soil through roots, plants can absorb Hg from the atmosphere through their stems and leaves. Studies of atmospheric mercury suggest that the leaves of the plant breathe through the pores and absorb the elemental Hg and methyl Hg in the atmosphere^27^. Various forms of atmospheric Hg can also be absorbed through wet and dry deposition into soil, and soil minerals and SOM also exhibit adsorption. Some plant enzymes are also capable of reducing plant uptake of organic mercury by converting it to inorganic mercury, which is then released into the atmosphere. A previous study^28^ showed that when vegetables were treated in low-pressure Hg similar to field gas Hg, the Hg content in the leaf parts of pepper was slightly higher than that in the roots. Therefore, relevant studies are needed on the effects of atmospheric Hg on the ability of vegetables to enrich Hg.

## Conclusions

Based on a meta-analysis of the role of soil Hg in vegetable absorption, enrichment coefficients of long beans, cowpea, and radish were found to be lower than the national standard for ES = ln BCF = −2.7076. The enrichment coefficient was higher in green pepper, spinach, cabbage, and Chinese cabbage.

Of five types of vegetables, the enrichment capacity of leaf was highest, and that of cucurbit was lowest. When soil pH was <6.5, Hg content was higher in vegetables. When soil pH was >7.5, Hg content was lower in vegetables. Therefore, in the cultivation of vegetables, if soil pH can be appropriately increased, reduced absorption of soil Hg by vegetables should occur.

When SOM is <20 g/kg, the enrichment ability of tuber of Hg is the highest, whereas that of eggplant is lower than that of other vegetables. When SOM is between 20 g/kg and 30 g/kg, the enrichment capacity of cucurbit is the lowest, whereas it is higher in leaf. When SOM is >30 g/kg, the adsorption capacity of the five vegetables is insignificant.

Through a statistical comparison of the soil enrichment capability for Hg in 24 types of vegetables, the results of the meta-analysis and the subgroup analysis were compared and analyzed. This provides useful information to aid in selecting and cultivating appropriate vegetable planting in Hg-polluted areas.

Various forms of Hg enter soil from the atmosphere through dry and wet deposition into the soil, and minerals and organic matter in the soil also play a role as adsorbers. Most Hg and its compounds are rapidly absorbed in SOM, with large concentrations of Hg remaining on the soil surface. A previous study^28^ showed that when a minimum amount of Hg (22.8 ng/m^3^) was used that was similar to field gas Hg content (13.5 ng/m^3^), the Hg content in pepper leaf parts was slightly higher than that in the root parts; this is also true for leafy vegetables such as cabbage, and for eggplant, the Hg levels of which were significantly higher than those in bean plants and cucurbit.

Phytoremediation, a novel and efficient green remediation technology, is an important means of controlling HM pollution in soil. There are many types of vegetables, and there are significant differences between crops in the absorption and accumulation of HMs. Selection of vegetable varieties with HM hyperaccumulation, or with the minimum capacity for Hg enrichment in soil, not only ensures in-depth study of absorption and transport mechanisms, but also aids in the cultivation of novel and useful vegetable varieties. It can also help cultivators to select plants suitable for different environmental conditions, enabling faster maturation and greater vegetable biomass.

Recent studies on the absorption and accumulation of HMs in vegetables have made some progress, but the physiological and molecular mechanisms remain unclear. Research on plant molecular mechanisms has found that to adapt to high concentrations of metal stress, plants form metal phytochelatins (PCs). When in excess, PCs play a key role in metal detoxification and maintenance of trace metal homeostasis. In addition, several types of metal transporters are involved in enrichment of HM ions, and the role of the genes associated with transport and accumulation of HMs in vegetables remains to be further studied.
